# Intraluminal Administration of Resiniferatoxin Protects against *Clostridium difficile* Toxin A-Induced Colitis

**DOI:** 10.1155/2017/8438172

**Published:** 2017-04-17

**Authors:** Steven R. Vigna

**Affiliations:** ^1^Departments of Cell Biology and Medicine, Duke University Medical Center, Durham, NC 27710, USA; ^2^V. A. Medical Center, Durham, NC 27705, USA

## Abstract

*Clostridium difficile* toxin A is a colonic inflammatory agent that acts partially by activation of TRPV1 (transient receptor potential vanilloid type 1). Resiniferatoxin (RTX) is an excitotoxin that activates TRPV1 at low concentrations and defunctionalizes TRPV1 at high concentrations. RTX at various doses was injected intraluminally into isolated ileal segments in anesthetized rats. After 3 hours, the treated segments were removed and inflammation was assessed. This acute treatment with RTX resulted in biphasic responses: (1) an increase in inflammation similar to that caused by toxin A and capsaicin at low doses of up to 100 ng RTX and (2) no inflammatory effect of RTX at higher doses (1–100 *μ*g), consistent with a defunctionalizing or neurotoxic effect of RTX at high doses. Separately, anesthetized rats were treated with RTX enemas and one or four weeks later were challenged with toxin A. Toxin A-induced colitis was significantly inhibited one week after an RTX enema, and this effect was RTX dose dependent. When tested four weeks after administration of the RTX enema, protection against toxin A colitis was lost. In conclusion, an RTX enema protects against toxin A-induced colitis in rats for at least one week but less than four weeks.

## 1. Introduction

*Clostridium difficile* is a noninvasive, anaerobic, spore-forming, gram-positive bacillus that is responsible for most cases of human infectious antibiotic-associated diarrhea and pseudomembranous colitis via production of exotoxins including toxin A [1]. The mechanisms by which toxin A causes colitis are incompletely known, but it has been shown that there is a neurogenic component involving the transient receptor potential vanilloid type 1 (TRPV1) cation channel expressed by a subset of primary sensory neurons [2–5]. There is evidence that toxin A in the lumen of the intestines causes the release of endogenous TRPV1 agonists [3, 6] that activate TRPV1 resulting in the release of proinflammatory neurotransmitters such as substance P (SP) [2] and subsequent acute inflammation. Evidence in support of this concept includes the demonstration that functional ablation of primary sensory neurons by systemic administration of high doses of the TRPV1 excitotoxin, capsaicin, for 3 consecutive days in adult rats strongly inhibited the inflammatory effects of toxin A [7]. In addition, pretreating rats systemically with a specific TRPV1 receptor antagonist, capsazepine, before administering toxin A significantly inhibited toxin A-induced inflammation; capsazepine alone had no effect [2]. Moreover, intraluminal administration of an exogenous TRPV1 agonist, capsaicin, caused structural damage to the ileum that was similar to that caused by toxin A, including complete loss of villi. Capsazepine pretreatment almost completely abolished the damaging effects of capsaicin [2]. These findings provide strong support for the hypothesis that toxin A causes neurogenic inflammation at least in part via activation of the TRPV1 receptor expressed by primary sensory neurons.

TRPV1 is activated by increased temperature, protons, and capsaicin [8], a vanilloid compound that is the pungent ingredient in chili peppers of the genus *Capsicum*, and by resiniferatoxin (RTX), an extract of the cactus-like plant *Euphorbia resinifera* [9]. The specific expression of TRPV1 by primary sensory neurons thus explains the excitotoxic effect of capsaicin and RTX administration—low concentrations or short exposure times activate TRPV1 transiently leading to increased cytoplasmic calcium concentrations and subsequent neurotransmitter release, whereas high concentrations or prolonged exposure times result in desensitization (defunctionalization) due to the excessive cytoplasmic concentrations of calcium or sodium ions resulting from TRPV1 activation [10].

These observations led us to reason that a potentially effective therapy for toxin A-induced colitis might be a local defunctionalization of TRPV1 by a TRPV1 excitotoxic drug such as capsaicin or RTX. Local rather than systemic TRPV1 defunctionalization is an important goal because whole body TRPV1 defunctionalization can result in severe and potential fatal side effects such as respiratory failure [9]. Both capsaicin and RTX have been shown to defunctionalize TRPV1 effectively in other systems [9, 11], but RTX has been shown to be both more potent than capsaicin and less irritating [9]. To achieve local rather than systemic defunctionalization of TRPV1, RTX was administered intraluminally into the colon in these studies.

## 2. Materials and Methods

### 2.1. Materials

Male Sprague-Dawley rats (175–200 g) were purchased from Charles River (Raleigh, NC). Resiniferatoxin (RTX) was purchased from Sigma (St. Louis, MO). *Clostridium difficile* toxin A was purchased from TechLab Inc. (Blacksburg, VA).

### 2.2. Surgery

Initial studies were performed in isolated ileal segments as described previously [2]. Briefly, animals were anesthetized with ketamine/xylazine (100/10 mg/kg, ip) and the ileum was exposed via a midline abdominal incision under sterile conditions. A 5 cm segment of the ileum was constructed by use of two ligatures 5 cm apart using a 4-0 silk suture. The lumen of the segment was then injected with 0.4 ml of either control vehicle solution (PBS) or RTX in various doses using a 26G, 3/8 in needle and 1 ml syringe. The ileum was kept moist with warm PBS throughout the 5 min procedure. Control rats were prepared identically, and their isolated ileal segments were injected with the PBS vehicle. The abdomen was closed in 2 layers (muscle and skin), and the animals were maintained anesthetized for 3 hours on a blanket warmed to 37°C. After 3 hours, the bowel segment was removed for further in vitro analysis and the animals were euthanized by anesthetic overdose (200 mg/kg nembutal, ip).

Colonic RTX enemas were administered to lightly sedated (ketamine/xylazine 40/10 mg/kg, ip) male rats (200 g body weight) in a volume of 0.5 ml injected with a 3 ml syringe connected to a rubber catheter followed by 1.5 ml of air. Control animals were treated with the vehicle (25% ethanol in PBS). The syringe was inverted so that fluid was injected into the colon before the air. The catheter was then inserted into the rectum to a length of 7 cm, and 0.5 ml of fluid was injected slowly while gently pinching the anus to prevent leakage, and then the additional 1.5 ml of air was slowly injected to ensure full delivery of the drug. The rats were then held upside down for at least 30 seconds to prevent loss of fluid and then returned to their cages with heads positioned below the anus to recover.

At either 1 week or 4 weeks after administration of the RTX enemas, isolated colonic segments were constructed as described above for ileal segments. A segment of the colon adjacent to the caecum was constructed by use of two ligatures 5 cm apart using a 4-0 silk suture. The lumen of the segment was then injected with 0.4 ml of either control vehicle solution (PBS) or toxin A (5 *μ*g) using a 26G, 3/8 inch needle and 1 ml syringe. The colon was kept moist with warm PBS throughout the 5 min procedure. The abdomen was closed in 2 layers (muscle and skin), and the animals were maintained anesthetized for 3 hours on a blanket warmed to 37°C. After 3 hours, the colonic segment was removed for further in vitro analysis and the animals were euthanized by anesthetic overdose (200 mg/kg nembutal, ip).

These studies were approved by the Durham VA and the Duke University Institutional Animal Care and Use Committees.

### 2.3. Luminal Fluid Accumulation

Luminal fluid accumulation was measured gravimetrically. After 3 hours of treatment, the isolated ileal segments were removed and weighed and their lengths were measured. Luminal fluid accumulation is expressed as mg wet weight per cm length.

### 2.4. Myeloperoxidase Activity

Myeloperoxidase (MPO) activity was measured as described previously [12]. Briefly, pieces of control and treated ileal segments were homogenized in 0.5% hexadecyltrimethylammonium bromide in 50 mM KH_2_P0_4_ (pH 6), freeze/thawed three times, and centrifuged at 4°C for 2 minutes, and then the absorbance of each supernatant was read at 460 nm at 0, 30, and 60 seconds after the addition of 2.9 ml of *o*-dianisidine dihydrochloride to 0.1 ml supernatant. The maximal change in absorbance per minute was used to calculate the units of MPO activity based on the molar absorbency index of oxidized *o*-dianisidine of 1.13 × 10^4^ M^−1^ cm^−1^. The results are expressed as MPO units of activity per gram of tissue wet weight.

### 2.5. Statistical Analysis

The results are expressed as mean ± SEM (*N* = 5–7). Mean differences among groups were assessed by one-way ANOVA and the Tukey-Kramer multiple comparison posttest using GraphPad Prism for Windows (GraphPad Software, San Diego, CA). In some cases when Bartlett's test revealed that the differences among the standard deviations of the various groups were significant, the data were analyzed by the Kruskal-Wallis nonparametric ANOVA followed by the Dunn's multiple comparison test. *P* values < 0.05 were considered significant. The numbers of rats per group are given in the figure legends.

## 3. Results

**Figure 1:**
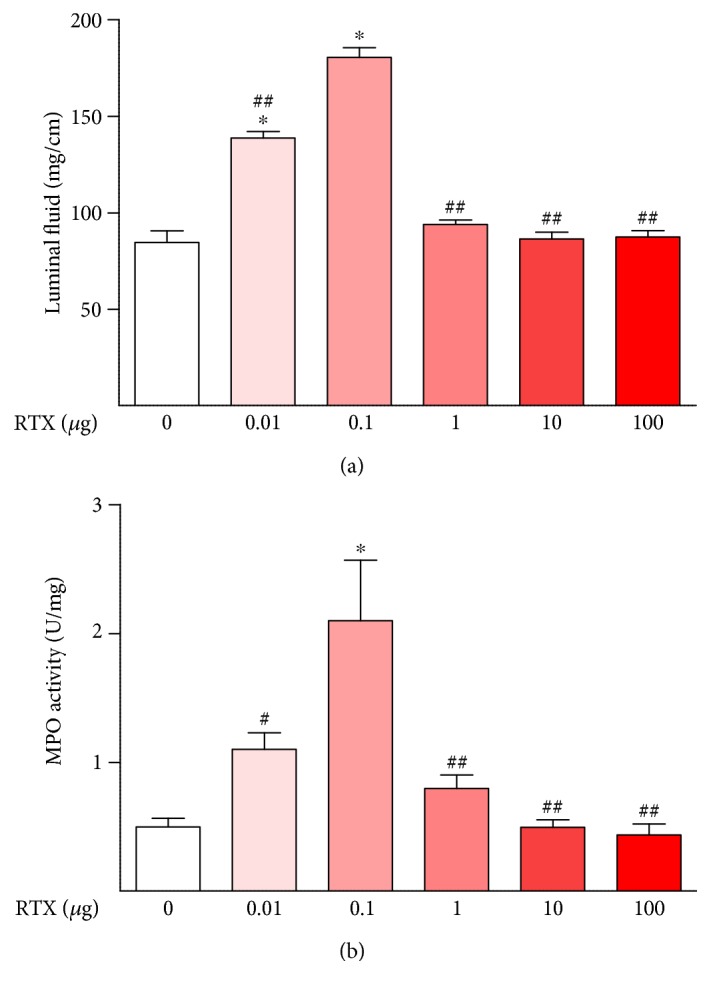
Dose-response effects of intraluminal RTX in the rat ileum. RTX had a biphasic inflammatory effect in this model. At low doses (0.01–0.1 *μ*g), RTX dose dependently caused increasing inflammation as assessed by luminal fluid accumulation and myeloperoxidase (MPO) activity. However, at higher doses (1–100 *μ*g), RTX had no effect on these inflammatory indices. ^∗^*P* < 0.001 versus 0 *μ*g RTX; ^#^*P* < 0.05 versus 0.1 *μ*g RTX; ^##^*P* < 0.001 versus 0.1 *μ*g RTX.

**Figure 2:**
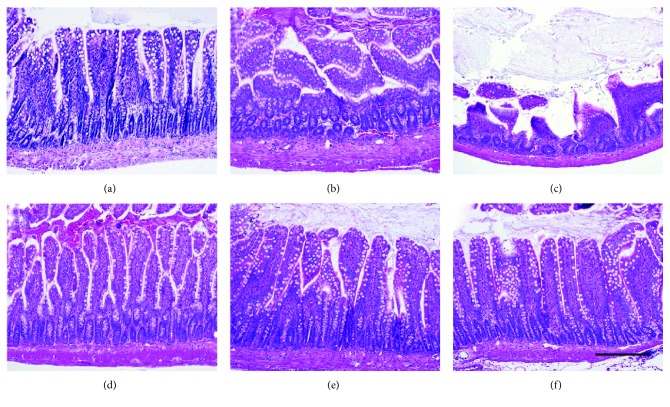
Dose-response effects of intraluminal RTX on ileal histopathology assessed by H&E staining. RTX had a biphasic effect on histopathology that matched the biphasic effects on luminal fluid accumulation and MPO activity seen in Figure 1. At low doses (0.01–0.1 *μ*g), RTX dose dependently caused increasing inflammation as assessed by loss of villi, loss of normal crypt architecture, and infiltration of immune cells into the lamina propria (panels (b) and (c)). However, at higher doses (1–100 *μ*g), RTX had no effect (panels (d)–(f)). (a) Vehicle control (no RTX); (b) 0.01 *μ*g RTX; (c) 0.1 *μ*g RTX; (d) 1.0 *μ*g RTX; (e) 10 *μ*g RTX; and (f) 100 *μ*g RTX. Scale bar = 100 *μ*m.

Intraluminal administration of RTX for 3 hours to isolated rat ileal segments at low doses (0.01–0.1 *μ*g) caused inflammatory responses including stimulation of luminal fluid accumulation (Figure 1(a)), increased tissue MPO levels (Figure 1(b)), and histopathology (Figure 2). The structural damage included virtually complete loss of villi, disrupted crypt architecture, hyperemia, and neutrophil infiltration (Figure 2). However, at higher doses (1–100 *μ*g), RTX did not have these effects (Figures 1 and 2), consistent with the well-known defunctionalizing effects of high doses of TRPV1 agonists, as discussed below.

**Figure 3:**
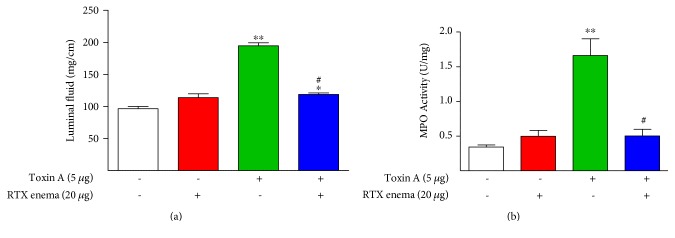
Effects of intraluminal toxin A (5 *μ*g) on colonic inflammation in rats given a single RTX enema at a defunctionalizing dose (20 *μ*g) administered 1 week before challenge with toxin A. Toxin A-induced luminal fluid accumulation (a) and MPO activity (b) were significantly inhibited by the prior RTX enema. ^∗^*P* < 0.05 versus toxin A−/RTX−; ^∗∗^*P* < 0.01 versus toxin A−/RTX−; ^#^*P* < 0.05 versus 5 *μ*g toxin A/RTX−.

**Figure 4:**
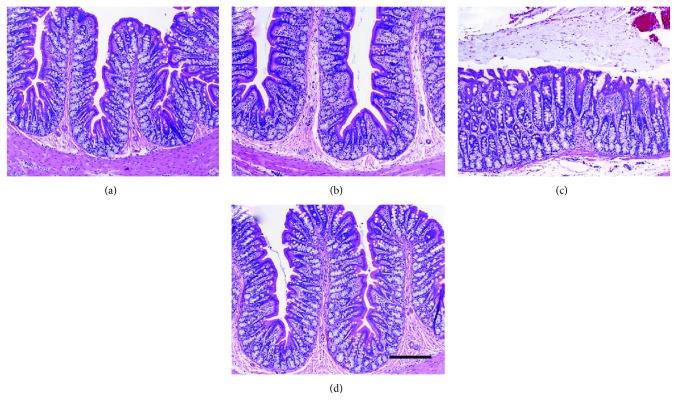
The protective effects of a single RTX enema at a defunctionalizing dose (20 *μ*g) administered one week before toxin A (5 *μ*g) on colonic histopathology in rats. (a) Normal colonic structure in vehicle controls; (b) normal colonic structure is also observed in rats given a 20 *μ*g enema of RTX one week before intraluminal vehicle treatment; (c) extensive colonic mucosal damage is observed in rats treated with 5 *μ*g of toxin A one week after a control vehicle enema, exhibiting loss of mucosal folding, loss of normal crypt architecture, and infiltration of immune cells into the lamina propria; and (d) virtually complete protection of colonic mucosal structure afforded by an RTX enema (20 *μ*g) given one week before toxin A administration (5 *μ*g). Scale bar = 100 *μ*m.

After showing that intraluminal RTX at high doses did not cause intestinal inflammation in the ileum, we asked whether a high dose of intraluminal RTX could protect the colon from *C. difficile* toxin A-induced acute inflammation, and if so, how long this protection would last. A 20 *μ*g RTX enema was administered one week prior to challenge with toxin A. We found that RTX given intraluminally one week before toxin A resulted in highly significant inhibition of toxin A-induced luminal fluid accumulation (Figure 3(a)), colonic MPO concentration (Figure 3(b)), and strong protection against toxin A-induced structural damage to the colon (Figure 4). The structural protection included preservation of mucosal folding, crypt architecture, and inhibition of neutrophil infiltration.

**Figure 5:**
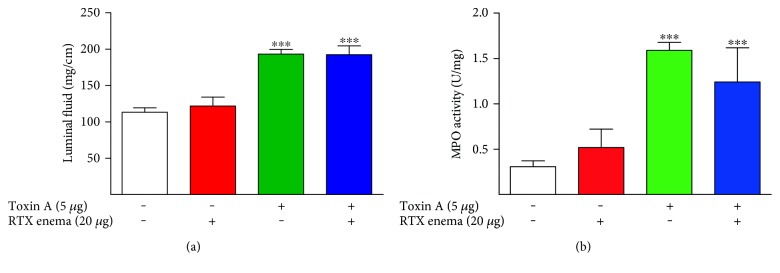
Effects of toxin A on colonic inflammation in rats given a single RTX enema administered 4 weeks before challenge with toxin A. Toxin A-induced luminal fluid accumulation (a) and MPO activity (b) were not significantly inhibited by an RTX enema given 4 weeks previously. ^∗∗∗^*P* < 0.001 versus toxin A−/RTX−.

**Figure 6:**
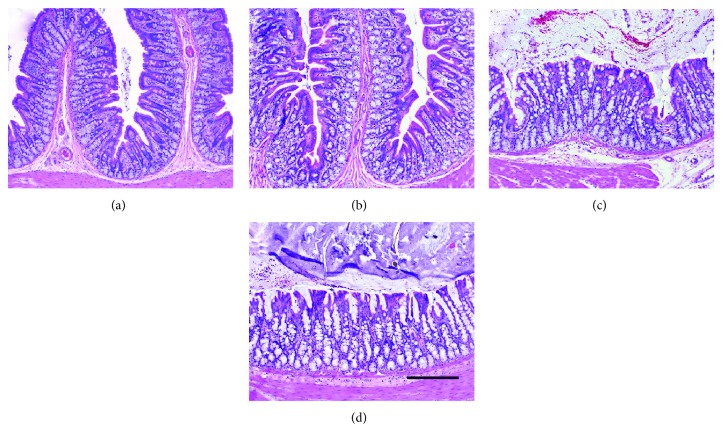
A single RTX enema (20 *μ*g) administered 4 weeks before intraluminal toxin A (5 *μ*g) does not protect colonic mucosal structure in rats. (a) Normal colonic structure in vehicle controls; (b) normal colonic structure is also observed in rats given a 20 *μ*g enema of RTX 4 weeks before intraluminal vehicle treatment; (c) extensive colonic mucosal damage is observed in rats treated with 5 *μ*g of toxin A 4 weeks after a control vehicle enema, exhibiting loss of mucosal folding, loss of normal crypt architecture, and infiltration of immune cells into the lamina propria; and (d) little to no protection of colonic mucosal structure is afforded by an RTX enema (20 *μ*g) given 4 weeks before toxin A administration (5 *μ*g). Scale bar = 100 *μ*m.

Based on these findings, we wondered how long the protective effects of a high dose of RTX given prophylactically as an enema would last in this model. To determine the duration of protection that RTX provides against toxin A colitis, we tested the effects of toxin A 4 weeks after a single RTX enema at a 20 *μ*g dose. We found that toxin A-induced luminal fluid accumulation (Figure 5(a)), increased MPO concentration (Figure 5(b)), and histopathology (Figure 6) were not inhibited 4 weeks after enema administration. Therefore, the protective effects of a 20 *μ*g RTX enema in this model last between 1 and 4 weeks.

**Figure 7:**
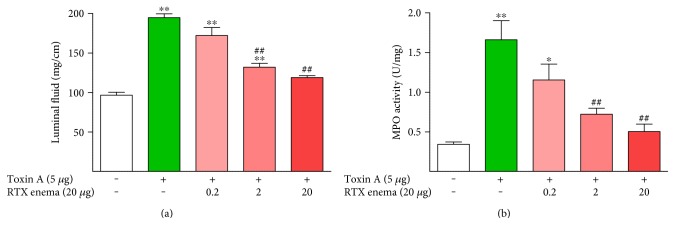
The protective effects of RTX enemas given one week before toxin A are dose dependent. ^∗^*P* < 0.05 versus toxin A−/RTX−; ^∗∗^*P* < 0.01 versus toxin A−/RTX−; ^##^*P* < 0.01 versus toxin A+/RTX−.

To determine if the protective effects of RTX enemas are dose responsive, various doses of RTX were administered as enemas one week before inflammation was induced by toxin A. RTX doses similar to those found to be noninflammatory in the ileum can also protect the colon from the damaging effects of toxin A in a dose-dependent manner (Figure 7). The maximal protective effect observed at the 20 *μ*g dose of RTX was in the range that did not cause inflammation when administered alone without toxin A for 3 hours in the previous experiment (Figures 1 and 2), but when administered as a pretreatment enema, it provided significant protection against toxin A colitis 1 week later.

## 4. Discussion

The damaging effects of RTX at low doses on the intestine are consistent with previous results obtained using acute intraluminal administration of another TRPV1 agonist, capsaicin [2]. Capsaicin caused a nearly identical pattern of villus loss, crypt disruption, and neutrophil infiltration as observed in the present study of the effects of RTX. In addition, the maximal effects of capsaicin were seen at a dose of 4 mg compared to the maximal damaging effect of RTX occurring at 0.1 *μ*g here, demonstrating that RTX is 4000 times more potent than capsaicin in this model, a difference that is consistent with the affinity of TRPV1 for these compounds [13]. In addition, the inflammatory effects of capsaicin and RTX in the ileum and colon are very similar to the effects of toxin A, further emphasizing the concept that endogenous TRPV1 agonists mediate some or all of the effects of *Clostridium difficile* toxin A on enteritis.

The damaging effects of RTX at low doses when given alone and the protective effects of the drug at higher doses when given before toxin A may seem peculiar. However, this pattern is consistent with the effects of TRPV1 agonists like RTX to stimulate the release of proinflammatory neurotransmitters from primary sensory neurons at low doses but to “desensitize” the neurons at higher doses before neurotransmitter release can occur [13]. This biphasic effect of TRPV1 agonists such as RTX has been termed excitotoxicity. Thus, RTX is an excitotoxin that stimulates TRPV1 at low doses and desensitizes it at high doses. The molecular mechanism of the “desensitizing” effects of TRPV1 agonists appears to involve excessive calcium and sodium ion influx into the neuron when high doses of agonists such as RTX activate the TRPV1 ion channel to open for long periods [14]. Although this lasting refractory state has traditionally been referred to as desensitization, the long-lasting absence of TRPV1 activation that occurs after high-dose or long-term stimulation differs considerably from true receptor desensitization [9]. Thus, this effect is not a true desensitization but can more accurately be termed a “defunctionalization” of the neuron resulting in inhibition of inflammation.

The mechanisms responsible for agonist-induced defunctionalization are not well understood. Local application of capsaicin or RTX to human skin or bladder (intravesicular) resulted in reductions in nerve fibers and loss of TRPV1 functions [15–19], suggesting that the defunctionalizing effect of the TRPV1 agonists was caused by loss of TRPV1-expressing primary sensory nerve fibers (axonal degeneration). The reductions in TRPV1 function returned over time and were accompanied by the reappearance of stainable markers for peripheral nerve fibers in these organs, demonstrating that the TRPV1-expressing neuronal cell bodies in the dorsal root ganglia (DRGs) were not abolished by these treatments. However, this interpretation may not be correct because these studies relied on immunohistochemical methods for staining neuronal markers, and a subsequent study demonstrated that the apparent axonal degeneration in the rat bladder after intravesicular capsaicin administration was not apparent using electron microscopy [20]. This finding suggests that the defunctionalizing effects of capsaicin and RTX may instead be due to axonal transport blockade that slows or stops the delivery of proinflammatory neurotransmitters such as substance P and CGRP to peripheral axons. It will require future experiments to test this hypothesis in the colon.

The present results demonstrate that protection against *C. difficile* toxin A-induced colitis lasts between one and four weeks in this model, but it is quite possible that longer treatments or higher doses of RTX may protect the colon for much longer intervals. For example, an 8% capsaicin skin patch applied for just 60 minutes to human volunteers caused reversible reduced epidermal nerve fiber concentrations and quantitative sensory nerve thresholds for between 1 and 12 weeks [21]. If TRPV1 defunctionalization protects the human colon from *C. difficile* toxin A colitis as it does in rats as shown here, this suggests a possible new therapy for the human disease caused by *C. difficile*. Resiniferatoxin could be administered to patients at doses and for intervals that result in maximum protection and fewest side effects. This therapy would be especially useful in treating patients with recurrent or intractable *C. difficile* colitis which is becoming increasingly prevalent worldwide [22, 23]. The drug could be given either as an enema or perhaps in pill form conjugated to itself or to an inactive molecule by an azo bond for specific release only in the presence of the azo bond-digesting bacteria in the colon in the same manner as the sulfasalazine-containing drugs used to treat ulcerative colitis and Crohn's disease [24]. It will be fascinating to observe future developments along these lines.
